# Physical and mental health impairments experienced by operating surgeons and camera-holder assistants during laparoscopic surgery: a cross-sectional survey

**DOI:** 10.3389/fpubh.2023.1264642

**Published:** 2023-09-07

**Authors:** Junjie Liu, Xi Qiao, Yi Xiao, Zhuofan Deng, Ji Cui, Mingdong Wu, Haolong Zhang, Kun Ran, Hailong Luo, Bo Tang

**Affiliations:** ^1^Vascular, Hernia & Abdominal Wall Surgery, The Second Affiliated Hospital of Chongqing Medical University, Chongqing, China; ^2^Precision Medicine Center, The Second Affiliated Hospital of Chongqing Medical University, Chongqing, China; ^3^Chongqing Municipality Clinical Research Center for Geriatrics and Gerontology, Chongqing, China; ^4^Gastrointestinal Surgery, The Second Affiliated Hospital of Chongqing Medical University, Chongqing, China; ^5^Hepatobiliary Surgery, The Second Affiliated Hospital of Chongqing Medical University, Chongqing, China; ^6^Obstetrics & Gynecology, The Second Affiliated Hospital of Chongqing Medical University, Chongqing, China; ^7^Hernia and Abdominal Wall Surgery, The Fourth Clinical College of Chongqing Medical University, Chongqing, China

**Keywords:** healthcare workers, work pressure, physical health, mental health, laparoscopic surgery

## Abstract

**Introduction:**

Surgeons may experience physical and mental health problems because of their jobs, which may lead to chronic muscle damage, burnout, or even withdrawal. However, these are often ignored *in camera*-holder assistants during laparoscopic surgery. We aimed to analyze the differences between operating surgeons and camera-holder assistants.

**Methods:**

From January 1, 2022, to December 31, 2022, a cross-sectional survey was conducted to evaluate the muscle pain, fatigue, verbal scolding, and task load for operating surgeons and camera-holder assistants. The Nordic Musculoskeletal Questionnaire, the Space Administration Task Load Index, and the Surgical Task Load Index (SURG-TLX) were combined in the questionnaire.

**Results:**

2,184 operations were performed by a total of 94 operating surgeons and 220 camera assistants. 81% of operating surgeons and 78% of camera-holder assistants reported muscle pain/discomfort during the procedure. The most affected anatomic region was the shoulders for operating surgeons, and the lower back for camera-holder assistants. Intraoperative fatigue was reported by 41.7% of operating surgeons and 51.7% of camera-holder assistants. 55.2% of camera-holder assistants reported verbal scolding from the operating surgeons, primarily attributed to lapses in laparoscope movement coordination. The SURG-TLX results showed that the distributions of mental, physical, and situational stress for operating surgeons and camera-holder assistants were comparable.

**Conclusion:**

Like operating surgeons, camera-holder assistants also face similar physical and mental health impairments while performing laparoscopic surgery. Improvements to the working conditions of the camera-holder assistant should not be overlooked.

## 1. Introduction

Laparoscopic surgery has gained increasing popularity and acceptance over the past few decades. It offers numerous advantages over traditional open surgery, including reduced postoperative pain, shorter hospital stays, quicker recovery times, and improved cosmetic outcomes (1). The technique involves the use of a laparoscope, a long, thin tube with a camera attached to it that allows surgeons to perform procedures through small incisions. The camera is controlled by the camera-holder assistant. The complex nature of laparoscopic surgery necessitates a coordinated effort between the operating surgeon and the camera-holder assistant to provide a steady view of the surgical field (2). The current research focused on improving the working environment of operating surgeons and reducing the damage to physical and mental health caused by laparoscopic surgery (3–8). However, the research on camera-holder assistants was limited.

The operating surgeon’s typical body position during the procedure is to stand upright with the arms suspended and extended for a considerable amount of time (9). To avoid affecting the surgical operation as much as possible, the camera-holder assistant often keeps the body posture of bending over and retracting the arms and sometimes even twists excessively (10). The prolonged maintenance of body postures can lead to an increased risk of musculoskeletal discomfort and fatigue (11–14). More than 70% of laparoscopic surgeons suffer from chronic muscle damage (15, 16). However, studies about intraoperative muscle pain with camera-holder assistants were limited. Therefore, we speculate that both the operating surgeon and the camera assistant will face physical challenges during laparoscopic surgery.

Beyond physical impairments, laparoscopic surgery may also confer mental impairments for both the operating surgeon and the camera assistant (17–20). Positive stress increases the surgeon’s alertness, resilience, motivation, and enjoyment of task completion (21). However, the impact of negative stress on surgical performance cannot be ignored. This source of mental stress may not be the same for the two roles. For the operating surgeon, this negative pressure comes from long-term operations, unsmooth operation procedures, changes in the difficulty of operations, and team cooperation (21). Sources of negative stress for camera-holder assistants might be related to operating surgeons in addition to surgical factors, such as verbal bullying. NUMANN et al. have shown that 47% of junior surgeons have suffered verbal bullying from senior surgeons during operations (22). Recent research has revealed that mental stress in the work environment for surgeons was associated with severe consequences, including burnout, withdrawal, and even suicide (23–25). However, mental health impairments experienced during laparoscopic surgery occur frequently among surgeons but are often underestimated among camera-holder assistants.

Surgery’s physical and mental effects on the operating surgeon have been widely researched, but the camera-holder assistant is often overlooked. The objective of our study was to analyze the variance in physical and mental impairments encountered by operating surgeons and camera-holder assistants during laparoscopic surgery.

## 2. Methods

### 2.1. Study design

In this cross-sectional survey, questionnaires were sent to all operating surgeons and camera-holder assistants who conducted laparoscopic inguinal hernia repair (LIHR), laparoscopic cholecystectomy (LC), laparoscopic appendectomy (LA), and laparoscopic ovarian cystectomy (LOC) from January 1, 2022, to December 31, 2022. Each of the four surgical types is performed by different departments. Specifically, an operating surgeon or camera-holder assistant only performs one surgical type. The questionnaire was sent to their electronic device 1 h after the operation. Before the survey, a statement was presented to inform participants that the purpose of the survey and that all data collected would be deidentified before analysis. And informed consent was automatically obtained from operating surgeons and camera-holder assistants who completed the questionnaire. The study has been approved by the ethics review committee of the Second Affiliated Hospital of Chongqing Medical University (Registration No. 2023100).

### 2.2. Inclusion and exclusion criteria

The inclusion criteria of the survey were as follows: (1) Both the operating doctor and the mirror assistant of the same operation completed this questionnaire and (2) All questions in the questionnaire were filled in completely. The exclusion criteria were as follows: (1) Questionnaires were answered more than 24 h after surgery, (2) The operating surgeon or camera-holder assistant was pregnant, and (3) The operating surgeon or camera-holder assistant has been changed during the operation.

### 2.3. Surgery and participant characteristics

We collected basic information about the operation, including the type of operation, operation time, the American Society of Anesthesiology (ASA) grade of the patient, emergency operation or not, and the number of consecutive operations in a single day by the participants. Furthermore, we also collected the demographics of the operating surgeons and the camera-holder assistants (e.g., sex, age, and medical history of chronic musculoskeletal conditions). To assess basic physical activity levels, we administered the International Physical Activity Questionnaire Long Form (IPAQ-LF) to participants on three separate occasions: January 1, July 1, and December 1, 2022 (26). The questionnaire comprised 27 questions, with a focus on physical activity in four different scenarios: work, transportation, daily life, and leisure. Based on the attributes of each physical activity and its metabolic equivalent (MET) assignment, we calculated the individual’s physical activity level over the past week. We then took the average of the three physical activity levels to represent the individual’s physical activity level for the year. Using the final scoring results, we categorized individuals into low, medium, and high levels of physical activity (Supplementary Appendix). Both the operating surgeon and the camera-holder are surgeons. According to our center’s practice, the surgeon performing the procedure possesses more surgical experience compared to the surgeon holding the camera (such as senior surgeons, residents, and trainees). It is important to note that there is no situation in which the senior surgeon acts as the camera-holder while the junior surgeon acts as the operating surgeon. However, there is a unique situation in which an experienced junior surgeon (who has served as a camera-holder assistant more than 100 times) assumes the role of the operating surgeon, while a less experienced junior surgeon serves as the camera holder. In these cases, the senior surgeon supervises the procedure without actively participating in it. If the junior surgeon encounters any difficulties during the procedure, the senior surgeon takes over. We excluded this situation. In our center, senior surgeons were defined as those who have independently performed over 100 laparoscopic surgeries.

### 2.4. The questionnaire

A comprehensive questionnaire was developed by combining the Nordic Musculoskeletal Questionnaire, the Space Administration Task Load Index (NASA-TLX), and the Surgical Task Load Index (SURG-TLX) (27–29). The questionnaire included three components: physical impairment, psychological impairment, and task load index (Supplementary Appendix).

A strict procedure was used to guarantee the confidentiality and anonymization of the survey questionnaire results. Personal identifying information was strictly excluded from the survey responses before analysis. Each questionnaire was assigned a unique identification code that was used to maintain confidentiality. During the data analysis phase, all identifying codes were detached from the dataset to make it impossible to link individual responses to specific participants. This deidentification process was performed to protect the privacy and anonymity of the survey participants. Access to the survey data was strictly limited to authorized personnel directly involved in the analysis and reporting. The participants were obligated by confidentiality agreements and ethical guidelines to uphold the privacy of the individuals involved and refrain from disclosing any identifiable information. Before the survey, a statement was presented to inform participants that the purpose of the survey was for research and that all data collected would be deidentified before analysis. There were no rewards or punishments offered to encourage or discourage participation.

The study included 9 questions related to body injuries, focusing on muscle pain experienced during and after surgery, including pain degree, pain location, and duration. The intensity of muscle pain was measured using the Visual Analog Scale (VAS pain) on a scale of 0–10 (30). Pain locations were categorized into several anatomic regions, including neck, shoulders, upper limbs (arms, wrists, and palms), upper back, lower back, hips/legs, knees, and ankles/feet. Participants were asked to select their first and second most impacted regions based on their intraoperative situation.

The mental health impact of the operation included 6 questions, which covered topics such as intraoperative and postoperative fatigue, verbal scolding from the operator to the camera-holder assistants during the operation, the reasons for the scolding, and whether it could cause psychological stress. Regarding scolding, the following potential reasons and others were identified in advance: poor coordination of laparoscopic lens movement, fatigue of the camera holder, operation error, and poor mood of the operating surgeon.

We incorporated schematic diagrams to visually score the surgical load. The surgical load was evaluated based on 6 aspects: mental demands, physical demands, temporal demands, task complexity, situational stress, and concentration degree. We scored on a scale of 0–20 based on their chosen location on the diagram. For example, a score of 0 in concentration indicated very low concentration, while a score of 20 indicated very high concentration.

### 2.5. The weighted scores of anatomic region votes

The weighted scores of options = (Σ Frequency × Weight) / Number, where the weight is determined by the position of the option in the ranking. For example, if there are 2 options involved in the ranking, the weight for the option in the first position is 2, and the second position is 1. The scores were then used to determine the most impacted anatomic regions for the operating surgeons and the camera-holder assistants.

### 2.6. Statistical analysis and approval

In this study, means and standard deviations were used for continuous variables. Medians and quarterback intervals were used for ordinal categorical variables, and constituent ratios were used for unordered categorical variables. To assess differences in unordered and ordered categorical variables, the chi-square test and the Mann–Whitney U test were used, respectively. *T*-tests were used to assess differences in continuous variables. SPSS software (SPSS 26.0, SPSS Inc., Chicago, Illinois) and R version 4.1.0 (R Foundation, 2021) were used for statistical analyses.

## 3. Results

Out of a total of 3,158 procedures, we received 2,447 (77.49%) and 2,826 (89.49%) questionnaire results from operating surgeons and camera-holder assistants, respectively. After applying the inclusion and exclusion criteria, we analyzed a total of 2,184 (69.16%) surgery questionnaires. Table 1 provided an overview of the basic characteristics of the procedures and demographic characteristics of the operating surgeons and the camera-holder assistants. The procedures included 187 LIHRs, 1,169 LCs, 450 LAs, and 378 LOCs.

**Table 1:** Characteristics of operations, operating surgeons, and camera-holder assistants.

Characteristics	Laparoscopic inguinal hernia repair (LIHR)	Laparoscopic cholecystectomy (LC)	Laparoscopic appendectomy (LA)	Laparoscopic ovarian cystectomy (LOC)
Number of operations, *n*	187	1,169	450	378
Number of surgeons, *n*				
Operating surgeons	13	28	30	23
Camera-holder assistants	34	58	78	50
Operation time, min, mean (SD)	112.7(46.9)	64.6 (26.0)	49.2 (25.7)	91.2 (39.5)
ASA, median (IQR)	2 (1)	2 (0)	2 (0)	2 (0)
Emergency surgery, *n* (%)	22 (11.8)	301 (25.7)	296 (65.8)	35 (9.3)
Consecutive operations^a^, *n*				
Operating surgeons				
One	174	834	409	334
Two or more	13	335	41	44
Camera-holder assistants				
One	178	903	440	355
Two or more	9	266	10	23
Sex, men, *n* (%)				
Operating surgeons	12 (92.3)	27 (96.4)	29 (96.7)	13 (56.5)
Camera-holder assistants	27 (79.4)	53 (91.4)	65 (83.3)	31 (37.25)
Age, years, mean (SD)				
Operating surgeons	37.6 (7.6)	38.2 (9.7)	36.5 (8.0)	37.4 (8.07)
Camera-holder assistants	26.8 (4.0)	26.1 (3.9)	25.6 (3.8)	27.1 (4.6)
Musculoskeletal disorders, *n* (%)				
Operating surgeons	7 (53.8)	15 (53.6)	14 (46.7)	10 (43.5)
Camera-holder assistants	8 (23.5)	11 (19.0)	11 (14.1)	7 (14.0)
IPAQ MET-min/w				
Operating surgeons	2575.4 (243.8)	2562.1 (113.9)	2542.2 (234.4)	2528.0 (273.6)
Camera-holder assistants	2533.4 (361.7)	2428.0 (262.8)	2404.5 (477.2)	2374.6 (477.2)

a Consecutive operations: the number of operations performed by a single individual on the same day before the survey operation.

SD, standard deviation; ASA, the American society of anesthesiologists; IQR, interquartile range; IPAQ, the international physical activity questionnaire; MET, metabolic equivalent.

The study involved a total of 94 operating surgeons, with 13 in LIHR, 28 in LC, 30 in LA, and 23 in LOC. Additionally, 220 camera-holder assistants participated, with 34 in LIHR, 58 in LC, 78 in LA, and 50 in LOC. Of the operating surgeons, 86.2% were men, while 80% of the camera-holder assistants were male. Chronic musculoskeletal disorders were present in 48.9% of operating surgeons compared to 16.8% of camera-holder assistants.

Results of the questionnaires were presented in Table 2. In 2184 laparoscopic surgeries, 81% of operating surgeons and 78% of camera-holder assistants experienced intraoperative muscle pain (*p* = 0.012). More than 70% of surgeons needed to adjust their posture during the procedure to alleviate pain. The VAS pain score of the operating surgeons was higher than that of the camera-holder assistants, and the difference was statistically significant (2.8 ± 1.8 vs. 2.4 ± 1.7, *p* < 0.001). After the operation, 73.2% of operating surgeons and 61% of camera-holder assistants reported persistent muscle pain after surgery (*p* < 0.001), with 13.3 and 20%, respectively, of them experiencing pain lasting more than 30 min. Half of the surgeons experienced fatigue during and after surgery. During the operation, fatigue was experienced in a lower proportion of operating surgeons than camera-holder assistants, while after the operation, the proportion was higher among operating doctors, and these differences were statistically significant (*p* < 0.001). Approximately 50.8% of the operating surgeons admitted to verbally scolding their camera-holder assistants during the operation, and this was significantly lower among camera-holder assistants. Most of the reasons for scolding were poor coordination of laparoscopic lens movement. However, approximately 64.6% of the camera-holder assistants reported experiencing mental pressure due to scolding.

**Table 2:** The results of the questionnaires of operating surgeons and camera-holder assistants.

	Operating surgeon (*N* = 2,184)	Camera-holder (*N* = 2,184)	*p*
Intraoperative pain/discomfort, *n* (%)			0.012
Never	416 (19.0)	481 (22)	
Occasionally	1,266 (58.0)	1,031 (47.2)	
Often	502 (23.0)	672 (30.8)	
Intraoperative posture adjusted			0.009
Never	541 (24.8)	611 (28.0)	
Occasionally	1,124 (51.5)	1,105 (50.6)	
Often	519 (23.8)	468 (21.4)	
VAS pain scores, mean (SD)	2.8 (1.8)	2.4 (1.7)	<0.001
Postoperative pain/discomfort, *n* (%)	1,598 (73.2)	1,333 (61.0)	<0.001
Duration of pain after surgery, *n* (%)			<0.001
0–10 min	1,008 (63.1)	696 (52.2)	
10–30 min	378 (23.7)	355 (26.6)	
>30 min	212 (13.3)	282 (21.2)	
Intraoperative fatigue, *n* (%)	910 (41.7)	1,127 (51.7)	<0.001
Postoperative fatigue, *n* (%)	1,132 (51.8)	1,008 (46.2)	<0.001
Verbal scolding^a^, *n* (%)	1,110 (50.8)	1,206 (55.2)	0.004
Reasons for the scolding, *n* (%)			0.034
Poor coordination of laparoscope movement	800 (72.1)	965 (80.0)	
Fatigue of camera holder	86 (7.7)	54 (4.5)	
Operation error	163 (14.7)	113 (9.4)	
Bad mood of the operating surgeon	31 (2.8)	8 (0.7)	
Others	30 (2.7)	66 (5.5)	
The mental stress of the scolding, *n* (%)	646 (58.2)	779 (64.6)	0.002

a The operating surgeon thought that the verbal scolding given to the camera-holder assistant during the operation/The camera-holder assistant thought that the verbal scolding received from the operating surgeon during the operation.

VAS, the visual analog scale; SD, standard deviation.

**Figure 1:**
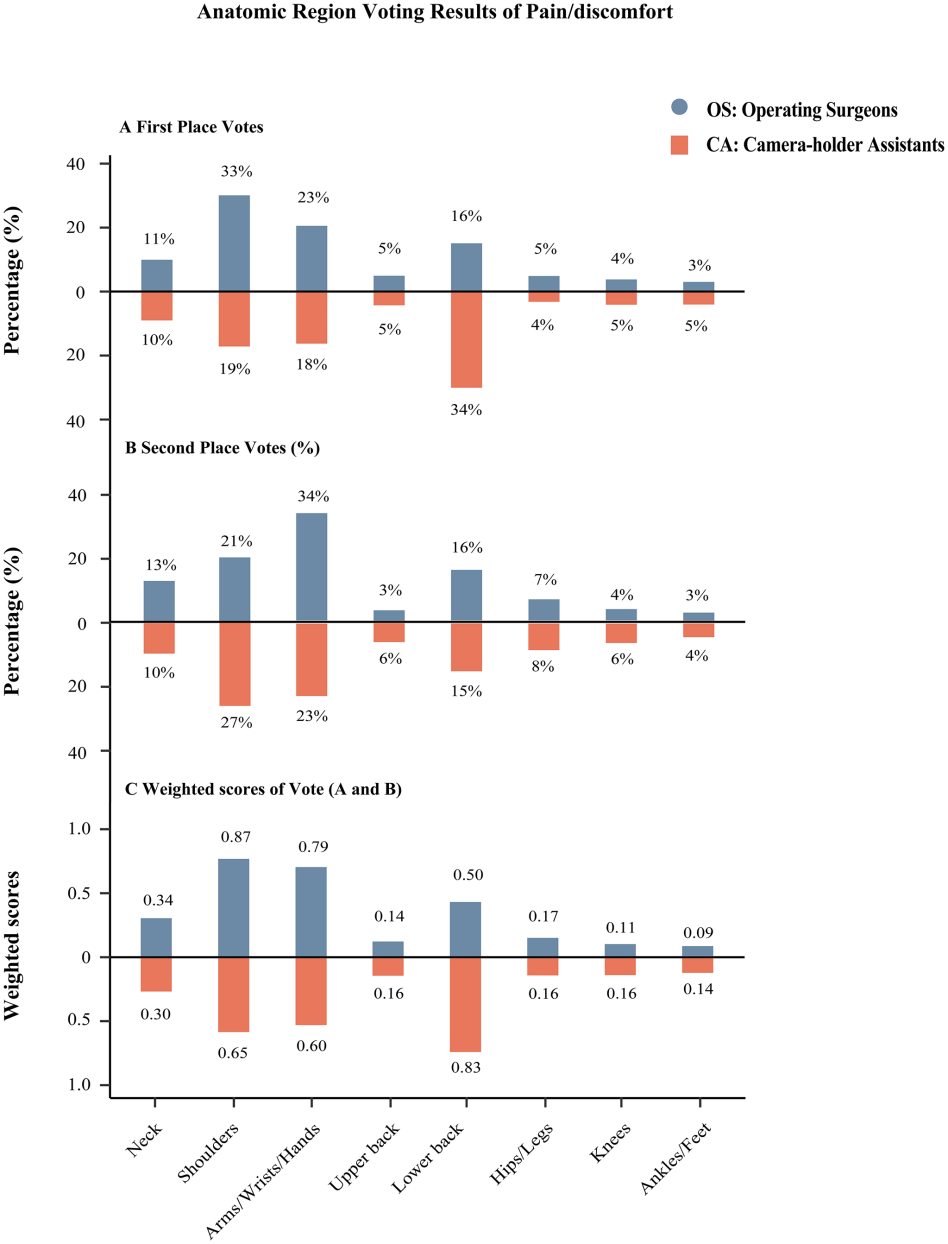
The results of the anatomic region of muscle pain/discomfort during the surgery. **(A)** The first vote of the anatomic muscle pain/discomfort region. **(B)** The second vote of the anatomic muscle pain/discomfort region. **(C)** The weighted scores of **(A,B)**.

The first and second vote anatomic region of muscle pain/discomfort during the operation and weighted statistical results were presented in Figure 1. The top three first-vote pain/discomfort anatomic regions for operating surgeons were shoulders, upper limbs (arms, wrists, palms), and lower back, whereas, for camera-holder assistants, they were lower back, shoulders, and upper limbs. In the second vote of pain/discomfort anatomic regions, upper limbs (arms, wrists, palms), shoulders, and lower back were the top three of the operating surgeons, while the camera-holder assistants chose shoulders, upper limbs (arms, wrists, palms), and lower back. After weighted scoring, the top three pain/discomfort anatomic regions affecting both the operating surgeons and the camera-holder assistant were the same, but the order was different. The operating surgeon’s shoulder was the most affected anatomic region, while the lower back was the most affected anatomic region for camera-holder assistants.

**Figure 2:**
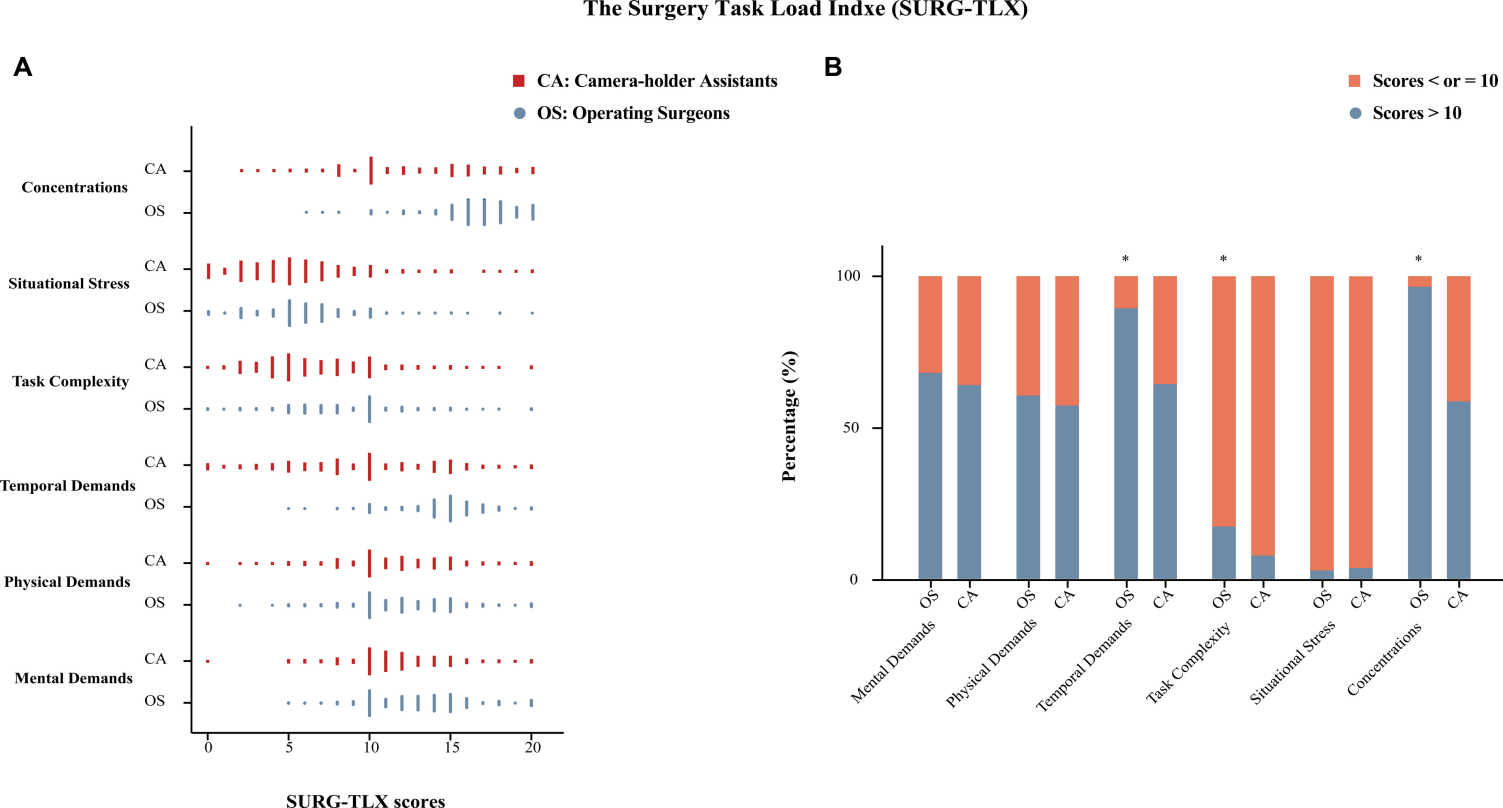
The results of the Surgery Task Load Index (SURG-TLX). **(A)** Scores (0–20) distribution of operating surgeons and camera-holder assistants in each part. **(B)** The proportion of operating surgeons and camera-holder assistants with scores ≤10 and > 10 in each part; *statistical difference.

Based on the results of SURG-TLX, the score distributions of mental demands, physical demands, and situational stress were similar between operating surgeons and camera-holder assistants. The scores were categorized into two groups based on scores >10 and ≤ 10. The proportion of these two groups was compared between operating surgeons and camera-holder assistants. The results indicated that there was no statistically significant difference in mental demands, physical demands, and situational stress between the two groups. More detailed results are presented in Figure 2.

## 4. Discussion

To the best of our knowledge, this was the first study to focus on the working environment of camera-holder assistants in laparoscopic surgery. In this cross-sectional survey, the mental and physical impairments experienced by the camera-holder assistant during the laparoscopic surgery should not be disregarded when compared to that of the operating surgeon. The results of SURG-TLX confirmed that there was no significant difference in the mental and physical impairments experienced by the camera-holder assistant and the operating surgeon. These findings highlight the importance of improving the working environment of camera-holder assistants in future research.

Stomberg et al. conducted a survey and found that over 70% of laparoscopic surgeons experienced symptoms related to musculoskeletal disorders, which was consistent with our study (15). However, the focus of their study did not include the working environment of camera-holder assistants. In our study, we found that over 70% of camera-holder assistants reported experiencing pain or discomfort during laparoscopic surgery, and most of them reported that these symptoms persisted after the surgery. According to our research, it has been found that 30.8% of camera-holder often experienced pain or discomfort during laparoscopic surgery, which was higher compared to operating surgeons (23%). Different postures during the operation may result in varying levels of pain or discomfort for the two roles. Furthermore, if the operating surgeon experiences any pain or discomfort, they have the flexibility to change their position, adjust their posture, or take breaks. However, the camera-holder faces challenges in relaxing as they need to maintain a stable field of view, even in uncomfortable situations. According to the SUR-TLX results, both camera-holder assistants and operating surgeons experienced similar levels of pain or discomfort during laparoscopic surgery. This indicated that both groups might be at risk of developing chronic musculoskeletal disorders (31).

Previous research has indicated that the predominant anatomic regions affected during laparoscopic surgery were the neck, lower back, shoulders, and upper limbs (3, 11, 15). Our study supported these findings and further identified differences between operating surgeons and camera-holder assistants. Weighted scores of votes revealed that the top three most affected anatomic regions among operating surgeons were the shoulders, upper limbs, and lower back. While the ranking was the lower back, shoulders, and upper limbs among camera-holder assistants. This difference might be related to the different body positions maintained during the procedure. Therefore, different ergonomic devices might be required to reduce physical health impairment during laparoscopic surgery for different roles. Currently, there was limited research on ergonomic equipment specifically designed for camera-holder assistants. Existing studies have focused on assistive support devices for operating surgeons (2, 6, 32–34). Therefore, it was crucial to conduct further research in this area to develop ergonomic solutions that could improve the working environment of camera-holder assistants.

In our study, camera-holder assistants were more likely to feel fatigue during surgery while operating surgeons reported a significantly higher incidence of fatigue after surgery. The exact reason was unclear, but we guess that it might be related to the level of surgical concentration. Our study showed that camera-holder assistants had significantly lower levels of concentration compared to operating surgeons. Research indicated that focused attention on a task could activate and invigorate the corresponding areas of the brain, so the operating surgeon was less likely to feel fatigued during the procedure (35). However, overexertion of concentration can lead to increased stress and exhaustion, ultimately diminishing brain function and performance (36).

It was worth noting that 55.2% of camera-holder assistants reported being verbally scolded by operating surgeons during surgery, which was significantly higher than reported by operating surgeons (50.8%). The operating surgeons might have underestimated the mental stress on the camera-holder assistant during the laparoscopic surgery through verbal scolding. Verbal scolding was frequently observed in the medical field (23, 25). Research has indicated that 47% of junior surgeons have experienced verbal scolding from senior surgeons while performing surgeries (22). The mental stress caused by verbal scolding could result in decreased motivation, termination from medical care, mental health problems, and even suicidal tendencies among camera-holder assistants. Junior surgeons frequently experience anxiety during high-pressure operations and might resort to using drugs to cope (37, 38). Reducing verbal scolding in the work environment by operating surgeons might be necessary to improve the work environment of camera-holder assistants.

Numerous studies have shown physical and mental impairments experienced by surgeons during surgery can affect surgical performance (13, 17, 19). We could also speculate that impairment of the physical and mental health of camera-holder assistants would also affect the performance of laparoscopic surgery. Prolonged holding of the laparoscope can cause hand muscle tremors, leading to lens instability and potentially increasing surgical risks. This influence might affect surgical performance through poor coordination of camera movement. Therefore, further research on the improvement of the work environment for camera-holder assistants both in terms of physical and mental health is necessary. Current research in robotic surgery, such as Da Vinci System (Intuitive Surgical, Sunnyvale, CA, United States), focused on improving the comfort of operating surgeons and reducing their ergonomic risks, making it highly praised (3, 13, 14). Moreover, there were robotic devices in development to replace camera-holder assistants. Examples of such devices include the HIWIN Robotic Endoscope Holder (HIWIN Technologies in Taichung, Taiwan, China) and the SOLOASSIST Robotic Camera Control (AKTORmed GmbH in Germany). However, traditional laparoscopic surgery remained the mainstream minimally invasive surgery worldwide, and the camera-holder assistant was a helpful role for junior surgeons to accelerate their learning curve in laparoscopic surgery and improve their understanding of laparoscopic techniques. Camera-holder assistants might not be replaced by machines for a long time. Designing an ergonomic device that matched the typical body posture of camera-holder assistants could be a potential solution at present.

Our study has several strengths. First, the large-scale questionnaire survey, combined with the rigorous procedure used in the retrieval process and data statistical analysis process, ensures the authenticity and reliability of the research results to the greatest extent possible. Additionally, our study is the first to examine the physical and psychological health impairments suffered by camera-holder assistants during laparoscopic surgery, an area that has been previously neglected. By shedding light on this issue, our study brings attention to the field and may help protect the careers of surgeons, particularly junior resident surgeons.

While our study has several advantages, we acknowledge some limitations. First, although we limited the return of questionnaires to within 24 h, recall bias might still be present, particularly for surgeons who perform multiple operations consecutively on the same day. Second, due to the single-center study, a total of 94 operating surgeons and 220 camera-holder assistants were responsible for the 2,184 operations. This could have introduced potential bias, as the same questionnaire was completed multiple times. Third, we intentionally did not define verbal blame, which might have resulted in differences in the perception of operating surgeons and camera-holder assistants. Our research results confirm this point, and it remains uncertain whether this cognitive difference overestimates or underestimates the verbal blame experienced during the actual procedure. Fourth, we did not include questions related to whether the surgeon had a mental illness. Some participants were reluctant to answer questions on this topic due to concerns about occupational risks. Therefore, we did not explore the effect of underlying mental illness in our study and could not determine whether this subgroup analysis was statistically significant. Fifth, this study was limited to disease diagnosis and treatment in our center; therefore, laparoscopic surgery for malignant diseases was not included in our study. Differences in surgical difficulty may have influenced our results.

## 5. Conclusion

The physical and mental health impairments experienced by camera-holder assistants during laparoscopic surgery should not be ignored. Prolonged operation time may have a greater impact on mental health. Improving working environment of camera-holder assistants to minimize physical and mental health impairment is conducive to improving the working interest, reducing job burnout, and delaying professional life.

### Consent to participate

Prior to the survey, a statement was presented to inform participants that the purpose of the survey was for research and that all data collected would be deidentified prior to analysis. And informed consent was automatically obtained from operating surgeons and camera-holder assistants who completed the questionnaire.

## Data availability statement

The raw data supporting the conclusions of this article will be made available by the authors, without undue reservation.

## Ethics statement

The studies involving humans were approved by the Bioethics Review Committee of the Second Affiliated Hospital of Chongqing Medical University. The studies were conducted in accordance with the local legislation and institutional requirements. Prior to the survey, a statement was presented to inform participants that the purpose of the survey was for research and that all data collected would be deidentified prior to analysis. And informed consent was automatically obtained from operating surgeons and camera-holder assistants who completed the questionnaire. The participants provided their written informed consent to participate in this study.

## Author contributions

JL: Conceptualization, Data curation, Investigation, Methodology, Project administration, Software, Writing – original draft, Writing – review & editing. XQ: Conceptualization, Data curation, Investigation, Methodology, Project administration, Software, Writing – original draft, Writing – review & editing. YX: Conceptualization, Data curation, Investigation, Methodology, Project administration, Writing – original draft. ZD: Conceptualization, Data curation, Investigation, Methodology, Project administration, Software, Writing – original draft. JC: Conceptualization, Data curation, Investigation, Methodology, Project administration, Software, Writing – original draft. MW: Conceptualization, Data curation, Investigation, Project administration, Writing – original draft. HZ: Conceptualization, Formal analysis, Investigation, Methodology, Project administration, Validation, Writing – original draft. KR: Conceptualization, Investigation, Methodology, Project administration, Writing – original draft. HL: Investigation, Methodology, Project administration, Writing – original draft. BT: Conceptualization, Funding acquisition, Investigation, Methodology, Project administration, Resources, Visualization, Writing – review & editing, Writing – original draft.

## Funding

The author(s) declare financial support was received for the research, authorship, and/or publication of this article. This work was supported by a grant from the National Natural Science Foundation of China (81400348) and the Kuanren Talents Program of the Second Affiliated Hospital of Chongqing Medical University.

## Conflict of interest

The authors declare that the research was conducted in the absence of any commercial or financial relationships that could be construed as a potential conflict of interest.

## Publisher’s note

All claims expressed in this article are solely those of the authors and do not necessarily represent those of their affiliated organizations, or those of the publisher, the editors and the reviewers. Any product that may be evaluated in this article, or claim that may be made by its manufacturer, is not guaranteed or endorsed by the publisher.
