# Supplementary material for: Physical and mental health impairments experienced by operating surgeons and camera-holder assistants during laparoscopic surgery: a cross-sectional survey
Source: Front Public Health. 2023 Sep 7;11:1264642. doi: 10.3389/fpubh.2023.1264642 (PMC10512950; doi:10.3389/fpubh.2023.1264642)
Supplement: Supplementary file 1 [file Table_1.DOCX]

**A. The Questionnaire**

**The First Part: Physical Impact**

| **Q1: Please indicate your role in the surgical team for this laparoscopic surgery?** | |
| --- | --- |
| 1. Operating Surgeon | ☐ |
| 1. Camera-holder assistant | ☐ |
| 1. Others Over | ☐ |

| **Q2: Do you currently have any chronic musculoskeletal disorders?** | |
| --- | --- |
| 1. Yes Q3 | ☐ |
| 1. No Q4 | ☐ |

| **Q3: If yes, what do you believe is the most likely cause of your chronic musculoskeletal disorder?** | |
| --- | --- |
| 1. Prolonged sitting next to a computer | ☐ |
| 1. Surgery | ☐ |
| c. Outside-work reasons | ☐ |

| **Q4: Do you experience muscle pain/discomfort during the surgery?** | |
| --- | --- |
| 1. Never Q8 | ☐ |
| 1. Occasionally Q5 | ☐ |
| 1. Often Q5 | ☐ |

| **Q5: Do you require adjusting your position during the surgery to alleviate this pain/discomfort?** | |
| --- | --- |
| 1. Never | ☐ |
| 1. Occasionally | ☐ |
| 1. Often | ☐ |

| **Q6: On a scale of 0-10, with 0 being no pain at all and 10 being unbearable severe pain, please rate the intensity of your pain.** |
| --- |
| 1. ______ |

| **Q7: Please select the anatomic regions you believe are most affected during the surgery, with the first affected anatomic region being the first-place vote and the second affected anatomic region being the second-place vote. Check the corresponding box next to each body part:** | |
| --- | --- |
| 1. Neck | ☐ |
| 1. Shoulders | ☐ |
| 1. Arms/Wrists/Hands | ☐ |
| 1. Upper back | ☐ |
| 1. Lower Back | ☐ |
| 1. Hips/Legs | ☐ |
| 1. Knees | ☐ |
| 1. Ankles/feet | ☐ |

| **Q8: Do you experience pain/comfort after the surgery?** | |
| --- | --- |
| 1. Yes | ☐ |
| 1. No Q10 | ☐ |

| **Q9: How long does this pain typically last?** | |
| --- | --- |
| 1. 0-10min | ☐ |
| 1. 10-30min | ☐ |
| 1. 30min- | ☐ |

**The Second Part: Mental Impact**

| **Q10: Do you experience fatigue during the surgery?** | |
| --- | --- |
| 1. Yes | ☐ |
| 1. No | ☐ |

| **Q11: Do you experience fatigue after the surgery?** | |
| --- | --- |
| 1. Yes | ☐ |
| 1. No | ☐ |

| **Q12: Have you ever experienced verbal abuse from the operating surgeon (Q1=b) /**  **have you verbally abused the assistant during the surgery (Q1=a)?** | |
| --- | --- |
| 1. Yes | ☐ |
| 1. No Q15 | ☐ |

| **Q13: What was the reason for the verbal abuse? Please select the relevant option(s).** | |
| --- | --- |
| 1. Poor coordination of laparoscopic lens movement | ☐ |
| 1. Fatigue of camera holder | ☐ |
| 1. Operation error | ☐ |
| 1. Bad mood of the operating surgeon | ☐ |
| 1. Others | ☐ |

| **Q14: Do you believe that such verbal abuse creates excessive mental stress for yourself (Q1=b)/puts excessive psychological pressure on the assistant (Q1=a)?** | |
| --- | --- |
| 1. Yes | ☐ |
| 1. No | ☐ |

**The Third Part: Surgical Task Load Index**

There are six rating scales that are meant for evaluating your experience during the procedure. Please evaluate the procedure by marking ‘‘X’’ one of the six scales at the point that best fits your experience. The scale ranges from ‘‘low’’ on the left

to ‘‘high’’ on the right. Please read the descriptions carefully.

| **Q15: Mental Demands**  **How mentally fatiguing were the procedures** |
| --- |
| 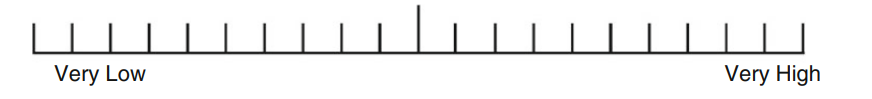 |

| **Q16: Physical Demands**  **How physically fatiguing was the procedure?** |
| --- |
| 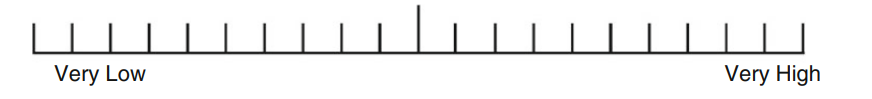 |

| **Q17: Temporal Demands**  **How hurried or rushed was the pace of the procedure?** |
| --- |
| 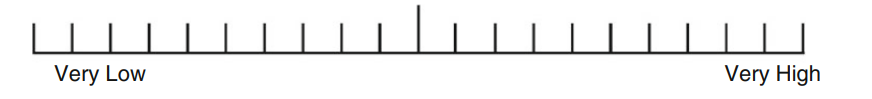 |

| **Q18: Task Complexity**  **How complex was the procedure?** |
| --- |
| 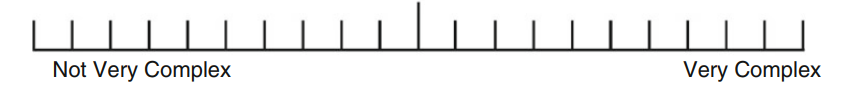 |

| **Q19: Situational Stress**  **How anxious did you feel while performing the procedure?** |
| --- |
| 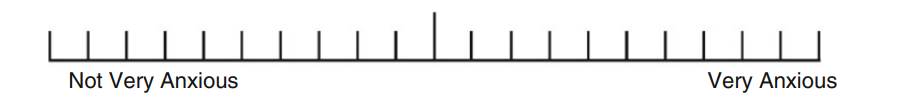 |

| **Q20:** **Concentrations**  **How Concentrations was the operating environment** |
| --- |
| 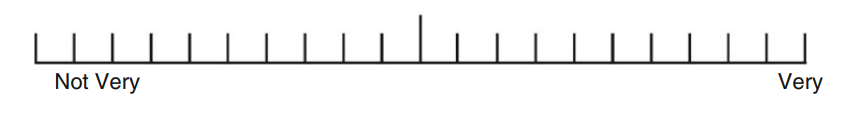 |

| **Q21: Please rate your operating surgeon/camera-holder assistant on a scale of 0-10, with 0 being highly dissatisfied and 10 being completely satisfied.** |
| --- |
| 1. ______ |

**B. Detailed explanations**

1. **IPAQ classification**

**International Physical Activity Questionnaire-Long Form (IPAQ-LF)** [**https://www.sralab.org/rehabilitation-measures/international-physical-activity-questionnaire-long-form**](https://www.sralab.org/rehabilitation-measures/international-physical-activity-questionnaire-long-form)

| **The amount of physical activity places the patient in 1 of 3 categories** | |
| --- | --- |
| **LOW** | **Do not meet criteria for categories Moderate or High** |
| **Moderate** | **Meet 1 the following:** |
|  | 1. **or more days with at least 20 minutes of vigorous activity** 2. **5 or more days with at least 30 minutes of moderate-intensity activity or walking** 3. **5 or more days of any combination of walking, moderate-intensity, or vigorous intensity activities with at least 600 MET-min/week** |
| **High** | **Meet 1 of the following:** |
|  | 1. **3 or more days of vigorous-intensity activity and at least 1500 MET-minutes/week** 2. **7 days of any combination of walking, moderate-intensity or vigorous intensity activities with a at least 3000 MET-minutes/week** |

1. **Relative position between operating surgeons and camera-holder assistants**

| **Ipsilateral** | | **Contralateral** |
| --- | --- | --- |
| **LIHR and LA** | **LC** | **LOC** |
| **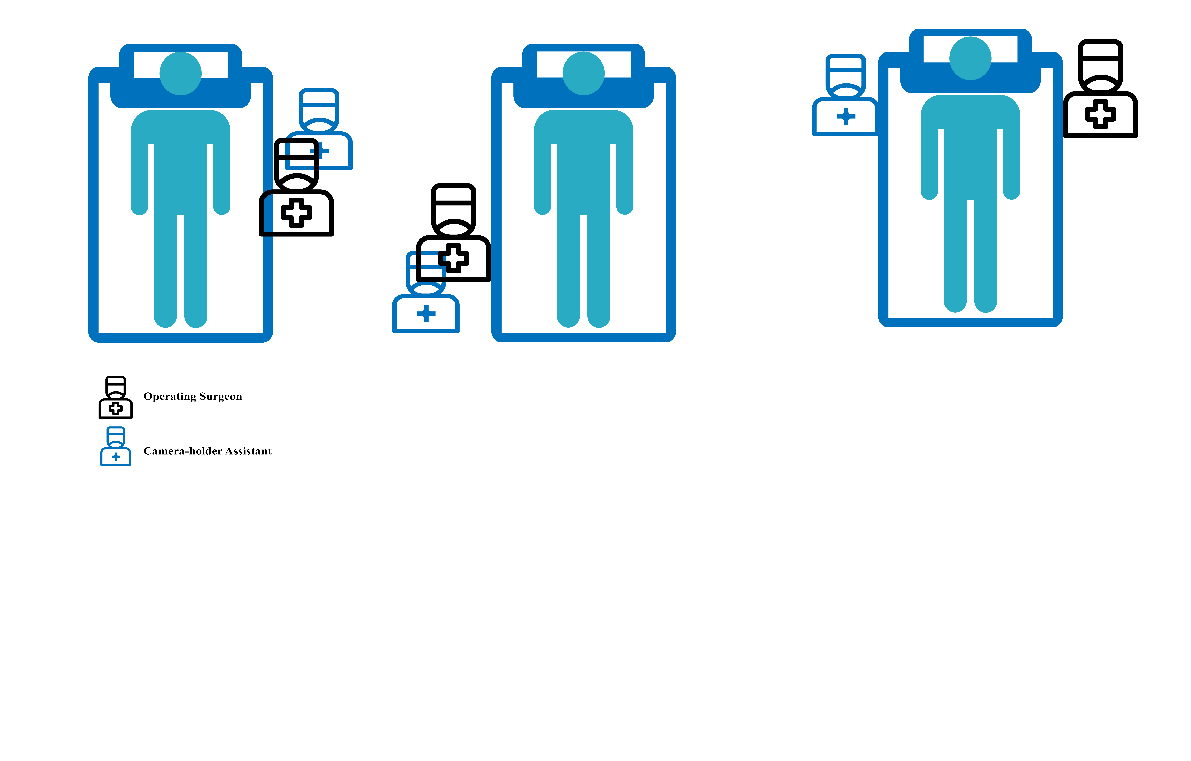** | | |

**laparoscopic inguinal hernia repair (LIHR), laparoscopic cholecystectomy (LC), laparoscopic appendectomy (LA), and laparoscopic ovarian cystectomy (LOC)**

**Copyright permissions:** All vectors and icons come from public relations open-source websites and have automatically obtained copyright permission.

**Vectors and icons by <a href="https://www.svgrepo.com" target="_blank">SVG Repo</a>**

1. **Surgical Experience**

| **Grading** | **Operating Surgeon** | **Camera-holder Assistant** |
| --- | --- | --- |
| Senior | One had experience with more than 100 laparoscopic surgeries. | Able to serve as a junior operating surgeon. |
| Junior | Not meet the above conditions | Not meet the above conditions |

**C. Supplement Statistical Analysis**

Table 1. The results of the questionnaires were obtained from participants who have experience working as both operating surgeons and camera-holder assistants.

|  | Operating surgeon  (N=1140) | Camera holder  (N=1160) | *P* |
| --- | --- | --- | --- |
| Intraoperative pain/discomfort, n (%) |  |  | 0.004 |
| Never | 230 (20.2) | 258 (22.1) |  |
| Occasionally | 668 (58.6) | 549 (47.0) |  |
| Often | 242 (21.2) | 361 (30.9) |  |
| Intraoperative posture adjusted |  |  | 0.026 |
| Never | 308 (27.0) | 326 (27.9) |  |
| Occasionally | 566 (49.6) | 582 (49.8) |  |
| Often | 266 (23.3) | 260 (22.3) |  |
| VAS pain scores, mean (SD) | 2.8 (1.8) | 2.4 (1.7) | <0.001 |
| Postoperative pain/discomfort, n (%) | 797 (69.6) | 728 (62.3) | <0.001 |
| Duration of pain after surgery, n (%) |  |  | <0.001 |
| 0-10 min | 497 (62.4) | 393 (54.0) |  |
| 10-30 min | 195 (24.5) | 201 (27.6) |  |
| >30 min | 105 (13.2) | 134 (18.4) |  |
| Intraoperative fatigue, n (%) | 476 (41.8) | 598 (51.2) | <0.001 |
| Postoperative fatigue, n (%) | 584 (51.2) | 519 (44.4) | 0.001 |
| Verbally Scolding, n (%) | 581 (51.0) | 617 (52.8) | 0.371 |
| The mental stress of the scolding, n (%) | 315 (54.2) | 416 (67.4) | 0.002 |
| **The Surgery Task Load Index (SURG-TLX),** mean (SD) |  |  |  |
| Mental demands | 13.2 (2.8) | 12.7 (2.7) | 0.413 |
| Physical Demands | 12.0 (3.1) | 11.6 (2.9) | 0.227 |
| Temporal demands | 14.6 (2.3) | 9.7 (4.4) | <0.001 |
| Task complexity | 8.9 (3.1) | 6.5 (3.2) | <0.001 |
| Situational stress | 5.5 (2.7) | 5.2 (3.4) | 0.241 |
| Concentration degree | 16.8 (2.1) | 12.8 (3.8 | <0.001 |
| **Pain/discomfort in anatomic regions** |  |  |  |
| Weighted scores of votes |  |  |  |
| Shoulders | 0.85 | 0.68 |  |
| Arms, Wrists, and Hands | 0.79 | 0.56 |  |
| Lower back | 0.52 | 0.81 |  |
